# *Candida kefyr* in Kuwait: Prevalence, antifungal drug susceptibility and genotypic heterogeneity

**DOI:** 10.1371/journal.pone.0240426

**Published:** 2020-10-27

**Authors:** Suhail Ahmad, Ziauddin Khan, Noura Al-Sweih, Wadha Alfouzan, Leena Joseph, Mohammad Asadzadeh

**Affiliations:** Department of Microbiology, Faculty of Medicine, Kuwait University, Jabriya, Kuwait; University of Arizona College of Medicine, UNITED STATES

## Abstract

**Objective:**

*Candida kefyr* causes invasive candidiasis in immunocompromised patients, particularly among those with oncohematological diseases. This study determined the prevalence of *C*. *kefyr* among yeast isolates collected during 2011–2018 in Kuwait. Antifungal susceptibility testing (AST) and genotypic heterogeneity among *C*. *kefyr* was also studied.

**Methods:**

Clinical *C*. *kefyr* isolates recovered from bloodstream and other specimens during 2011 to 2018 were retrospectively analyzed. All *C*. *kefyr* isolates were identified by CHROMagar Candida, Vitek2 and PCR amplification of rDNA. AST was performed by Etest. Molecular basis of resistance to fluconazole and echinocandins was studied by PCR-sequencing of *ERG11* and *FKS1*, respectively. Genotypic heterogeneity was determined with microsatellite-/minisatellite-based primers and for 27 selected isolates by PCR-sequencing of IGS1 region of rDNA.

**Results:**

Among 8257 yeast strains, 69 *C*. *kefyr* (including four bloodstream) isolates were detected by phenotypic and molecular methods. Isolation from urine and respiratory samples from female and male patients was significantly different (*P* = 0.001). Four isolates showed reduced susceptibility to amphotericin B and one isolate to all (amphotericin B, fluconazole, voriconazole and caspofungin/micafungin) antifungals tested. Fluconazole-resistant isolate contained only synonymous mutations in *ERG11*. Echinocandin-resistant isolate contained wild-type hotspot-1 and hotspot-2 of *FKS1*. Fingerprinting with microsatellite-/minisatellite-based primers identified only three types. IGS1 sequencing identified seven haplotypes among 27 selected isolates.

**Conclusions:**

The overall prevalence of *C*. *kefyr* among clinical yeast isolates and among candidemia cases was recorded as 0.83% and 0.32%, respectively. The frequency of isolation of *C*. *kefyr* from bloodstream and other invasive samples was stable during the study period. The *C*. *kefyr* isolates grown from invasive (bloodstream, bronchoalveolar lavage, abdominal drain fluid, peritonial fluid and gastric fluid) samples and amphotericin B-resistant isolates were genotypically heterogeneous strains.

## Introduction

*Candida* and some other yeast species colonize humans during or soon after birth and form part of normal microbial flora of mucosal surfaces of the gastrointestinal/genitourinary tracts and skin [1–3]. The isolation of *Candida* and other yeast species is usually higher from individuals receiving broad-spectrum antibiotics or corticosteroid treatment or from individuals with other debilitating underlying conditions that compromise/reduce host immunity such as diabetes, cancer, extremes of age (neonates and elderly), pregnancy and human immunodeficiency virus infection [3–5]. Typically, these conditions also predispose the colonized individuals to invasive or mucocutaneous infections by *Candida* or other yeast species [3–6]. Although *Candida albicans* is the most common cause of candidemia/invasive candidiasis, >50% of all *Candida* infections are now caused by other non-*albicans* species of *Candida* and are usually associated with high mortality rates [7–10]. In recent years, increasing number of reports have described invasive infections by drug-resistant/multidrug-resistant *Candida* spp. in medical centers worldwide [11–14]. The emerging multidrug-resistant *Candida* spp. include *Candida auris*, *Candida haemulonii* complex members, *Candida glabrata*, *Candida guilliiermondii* complex members, *Candida krusei*, *Candida lusitaniae*, *Candida lipolytica*, *Candida rugosa* and *Candida kefyr* [11–15].

*Candida kefyr* (*Kluyveromyces marxianus*), an ascomycetous yeast occasionally isolated from dairy products [16, 17], has also been isolated from a variety of clinical specimens including invasive samples and from the hands of health care workers [14, 18–21]. Recent reports suggest that *C*. *kefyr* is an emerging pathogen in immunocompromised patients, particularly those with oncohematological diseases [14, 22–25]. *C*. *kefyr* has attracted attention due to its reduced susceptibility to amphotericin B [24, 26–29] and its ability to acquire resistance to echinocandins rapidly [30, 31]. The epidemiology of *C*. *kefyr* as a human pathogen is poorly understood due to lack of well-defined studies, particularly from the Middle Eastern countries. Here, we describe molecular characterization, susceptibility to antifungal agents and genotypic heterogeneity among a large collection of *C*. *kefyr* isolates collected from various clinical specimens over an 8-year period in Kuwait.

## Materials and methods

### Reference strains and clinical yeast isolates

Reference strains or well characterized clinical isolates of *C*. *kefyr* (ATCC28838, ATCC26548 and CBS4857), *C*. *albicans* (ATCC90028), *Candida parapsilosis* (ATCC22019), *C*. *glabrata* sensu stricto (CBS138), *Candida nivariensis* (CBS9983), *Candida bracarensis* (CBS10154), *Candida tropicalis* (ATCC750), *Candida dubliniensis* (CBS7987), *C*. *lusitaniae* (CBS4413), *C*. *guilliermondii* (CBS6021) and *Candida famata* (CBS796) were used as reference *Candida* species. The clinical specimens for this retrospective study were collected from adult patients after obtaining informed verbal consent only at nine major public sector hospitals spread out across Kuwait for identification and antifungal susceptibility testing (AST) of fungi as part of routine patient care and diagnostic work-up and the data are reported on deidentified samples from each patient. The study and the consent procedure were approved by the Ethical Committee of Health Sciences Center, Kuwait University (Approval no. VDR/EC/2477). All clinical yeast isolates were streaked on Sabouraud dextrose agar (SDA) (Difco) supplemented with chloramphenicol (50 mg/L) plates for checking purity before phenotypic and molecular identification studies.

### Phenotypic and molecular identification

All yeast isolates were processed for identification by colony characteristics on CHROMagar Candida and by Vitek2 yeast identification system (bioMérieux, Marcy-L´Etoile, France) as described previously [32]. Bloodstream isolates were also tested by matrix-assisted laser desorption/ionization time-of-flight mass spectrometry (MALDI-TOF MS; bioMérieux, Marcy l’Etoile, France). Briefly, a single colony from a fresh culture of the isolate on SDA was suspended in 1 ml of 70% ethanol, vortexed briefly, and centrifuged at 13,000xg for 2 minutes in a microfuge. The pellet was re-suspended in 50 μl of 70% formic acid (Fluka, USA) and 50 μl of acetonitrile (Fluka, USA), vortexed briefly and centrifuged for 2 minutes at 13,000xg. One μl of the extracted supernatant was transferred to an individual spot on the 48 well VITEK MS disposable target slide, covered with 1 μl ready to use VITEK MS HCCH matrix (bioMerieux) and air dried. The slides were processed by VITEK MS machine and the data were interpreted according to manufacturer’s instructions and as described in detail previously [33].

Molecular identity of each *C*. *kefyr* isolate was established by developing a simple species-specific PCR assay targeting the internal transcribed spacer (ITS) region of rDNA. DNA from reference strains and clinical yeast isolates was extracted by the rapid boiling method using Chelex-100 as described previously [34] or by using Gentra Puregene Yeast DNA extraction kit (Qiagen, Hilden, Germany) used according to kit instructions. The ITS region was amplified by using *C*. *kefyr*-specific forward (CKEF, 5’-GCTCGTCTCTCCAGTGGACATA-3’) and reverse (CKER, 5’-ACTCACTACCAAACCCAAAGGT-3’) primers by using the reaction and cycling conditions and amplicons were detected by agarose gel electrophoresis, as described previously [34]. The species specificity of the combination of CKEF and CKER primers for *C*. *kefyr* was indicated by BLAST searches (http://blast.ncbi.nlm.nih.gov/Blast.cgi?). The PCR assay should yield an amplicon of 268 bp from *C*. *kefyr* while no amplicon is expected from other *Candida* or other yeast species. The identification of 27 selected isolates was also confirmed by PCR-sequencing of the ITS region of rDNA by using panfungal primers, as described previously [35]. BLAST searches (http://blast.ncbi.nlm.nih.gov/Blast.cgi?) were performed and >99% sequence identity was used for species identification [36].

### Antifungal susceptibility testing

The AST was performed by Etest (bioMérieux SA, Marcy-l’-Etoile, France) according to manufacturer's instructions and as described previously [37]. Reference strains of *C*. *parapsilosis* (ATCC22019) and *C*. *albicans* (ATCC90028) were used for quality control. Since there are no susceptibility breakpoints available for *C*. *kefyr*, the isolates were described as susceptible, intermediate/susceptible dose-dependent and resistant using Clinical and Laboratory Standards Institute (CLSI) breakpoints used for *C*. *albicans* as follows: <2, 4 and >8 μg/ml for fluconazole, <0.12, 0.25–0.5, and >1 μg/ml for voriconazole and <0.25, 0.5 and >1 μg/ml for caspofungin, respectively [38]. Although there is no accepted clinical breakpoint, the isolates with minimum inhibitory concentrations (MICs) of >1 μg/ml for amphotericin B were considered as non-wild-type (resistant) [24].

### PCR-sequencing of *ERG11* gene for fluconazole resistance

The *ERG11* gene was amplified as two overlapping fragments by using *C*. *kefyr*-specific amplification primers by using the *ERG11* sequence from *C*. *kefyr* ATCC26548 (GenBank accession no. KF964546) and CBS4857 (GenBank accession no. CP015055) as reference. *C*. *kefyr* ATCC26548 (= CBS6556) is susceptible to triazoles (fluconazole, voriconazole and itraconazole), echinocandins (caspofungin and micafungin) and amphotericin B [39]. The N-terminal fragment was amplified by using CkefERG11F1 (5’-GAGAATTGGCGATACAGACTAA-3’) and CkefERG11R1 (5’-TTATCRGTCATCTTAGTACCATC-3’) primers and C-terminal fragment was amplified by using CkefERG11F2 (5’-GGTTTCACTCCATTGAACTTCGT-3’) and CkefERG11R2 (5’-GTAAAACTTGTCGGAGGGAAGAA-3’) primers. Other reaction conditions and cycling parameters were same as described previously for PCR amplification of *ERG11* gene from *C*. *parapsilosis* [40]. The amplicons were sequenced in both directions by using the DNA sequencing protocol for *C*. *parapsilosis ERG11* gene as described previously [40] except that *C*. *kefyr*-specific primers listed below were used. N-terminal amplicons were sequenced with CkefERG11FS1 (5’-ATTGGCGATACAGACTAAGAATA-3’), CkefERG11FS2 (5’-GTACTTGGGGCCAAAGGGTCACGA-3’) or CkefERG11RS1 (5’-AACGAACTTCTTTTGGTCCATTAG-3’) or CkefERG11RS2 (5’-GTCATCTTAGTACCATCYTTGTA-3’) primer. C-terminal amplicons were sequenced with CkefERG11FS3 (5’-CTATCGTAAGAGAGACCATGCCCA-3’) or CkefERG11FS4 (5’-TTGCACTCTTTGTTCCGTAAAGT-3’) or CkefERG11RS3 (5’-TCTTGCAAATGACAGTAACCTGG-3’) or CkefERG11RS4 (5’-AAACTTGTCGGAGGGAAGAAAATA-3’) primer. The complete *ERG11* sequences of 1752 bp were assembled and were compared with the corresponding sequences from reference *C*. *kefyr* strains ATCC26548 and CBS4857 by using Clustal omega (https://www.ebi.ac.uk/Tools/msa/clustalo/).

### PCR-sequencing of hotspot-1 and hotspot-2 of *FKS1* gene for echinocandin resistance

The mutations conferring resistance to echinocandins typically are located in hotspot-1 or hotspot-2 region of *FKS1* gene [14, 31, 41]. The hotspot-1 region of *FKS1* gene was amplified by using *C*. *kefyr*-specific CkefFKS1F1 (5’-GGTCTTGATATGTGGATGTCCTA-3’) and CkefFKS1R1 (5’-AAATGTTTCTCCATGGAGTCAAA-3’) primers while hotspot-2 region was amplified by using *C*. *kefyr*-specific CkefFKS1F2 (5’-TGGGTACACAATTGCCACTTGA-3’) and CkefFKS1R2 (5’-AATATAACGAGCACCACCGATA-3’) primers. Other reaction and cycling conditions were same as described previously for the amplification of *FKS1* gene from *C*. *tropicalis* [42]. Both strands of purified amplicons were sequenced with internal *C*. *kefyr*-specific sequencing primers for hotspot-1 (CkefFKS1F1S, 5’-CTTGATATGTGGATGTCCTACTT-3’ or CkefFKS1R1S, 5’-TGTTTCTCCATGGAGTCAAAATG-3’) and for hotspot-2 (CkefFKS1F2S, 5’-TACACAATTGCCACTTGACCGT-3’ or CkefFKS1R2S, 5’-TAACGAGCACCACCGATAGTTA-3’) by following the DNA sequencing protocol as described previously [42].

### Molecular fingerprinting studies

The genotypic heterogeneity among *C*. *kefyr* isolates was investigated by using minisatellite-based (M13-MIN, 5’-GAGGGTGGCGGTTCT-3’) and microsatellite-based (GACA_4_, 5’-GACAGACAGACAGACA-3’) primers, as described previously [43]. Additional fingerprinting for 27 selected *C*. *kefyr* isolates was performed by PCR-sequencing of the non-transcribed intergenic spacer (IGS)-1 region located between 28S rRNA and 5S rRNA genes in rDNA. The IGS-1 was amplified by using panfungal NTS1F (5’-GGGATAAATCATTTGTATACGAC-3’) and NTS1R (5’-TTGCGGCCATATCCACAAGAAA-3’) primers and the PCR amplification reaction and cycling conditions as described previously [44]. The amplicons were purified and both strands were sequenced as described previously [44] except that NTS1FS (5’-CGGAGTATTGTAAGCAGTAGA-3’), CkefNTS1FS2 (5’-GCCATGTAAATACGTCTTCGA-3’), CkefNTS1RS1 (5’-TGCTATAGGATAGTACTGCAGC-3’) or CkefNTS1RS2 (5’-GCATGCACATAAGTAATGTGA-3’) was used as sequencing primer. The IGS-1 sequences for each isolate were assembled. The phylogenetic tree was constructed by using BioNumerics v7.5 software (Applied Maths, Sint-Martens-Latem, Belgium) and standard unweighted pair group method with arithmetic mean (UPGMA) settings. The robustness of tree branches was assessed by bootstrap analysis with 1,000 replicates.

### Statistical analysis

Statistical analysis was performed by using Fisher’s exact test or chi-square test as appropriate and probability levels <0.05 by the two-tailed test were considered as significant. Statistical analyses were performed by using WinPepi software ver. 11.65 (PEPI for Windows, Microsoft Inc., Redmond, WA, USA).

## Results

### Prevalence of *C*. *kefyr* among yeast isolates and phenotypic and molecular identification

Of 8257 yeast isolates from same number of patients tested during the 8-year study period (2011 to 2018), 69 isolates from 69 patients (only one isolate from each patient was considered) were identified as *C*. *kefyr* with an overall prevalence of 0.83% among total yeast species isolates (Table 1). Repeat isolates were also obtained from 11 patients. Four bloodstream and seven isolates from other invasive (such as bronchoalveolar lavage, abdominal drain fluid, peritonial fluid and gastric fluid) samples were included among 69 *C*. *kefyr* (Table 1). The remaining 58 isolates were obtained from non-invasive (such as urine, sputum, tracheal aspirate, vaginal swab, throat swab, upper palate swab and ear swab) were considered as non-invasive samples. One bloodstream isolate came from a patient with acute lymphocytic leukemia. The occurrence of total *C*. *kefyr* isolates and isolates from bloodstream and other invasive samples during the two 4-year-periods (2011 to 2014 and 2015–2018) was nearly same (Table 1). A total of 1238 bloodstream isolates were recovered from 1238 candidemia patients during the same study period. Thus, the prevalence of *C*. *kefyr* among bloodstream *Candida* spp. isolates was low (4 of 1238, 0.3%). The clinical details, history of treatment with antifungal drugs and outcome were available for 2 of 4 patients with candidemia. Although both isolates (Kw1609/11 and Kw3267/11) were susceptible to all four antifungal drugs, Patient 1 treated with fluconazole for 21 days and Patient 2 treated with caspofungin for 1 month died.

**Table 1:** Distribution of total, invasive and amphotericin B (AMB)-resistant *C*. *kefyr* strains detected among clinical yeast isolates collected during 2011–2018 in Kuwait.

Year of	No. of yeast	No. of *C*. *kefyr*	No. of *C*. *kefyr* from	No. of AMB-resistant
isolation	isolates tested	isolates detected	invasive samples	*C*. *kefyr* isolates
2011	926	11	2	1
2012	924	5	0	0
2013	1052	9	1	0
2014	869	8	2	0
2015	1068	10	0	1
2016	1196	10	1	0
2017	1033	7	1	1
2018	1189	9	4	2*
**Total**	**8257**	**69**	**11**	**5**

*One isolate (Kw2153/18) was resistant to amphotericin B, fluconazole, voriconazole, caspofungin and micafungin.

The distribution of *C*. *kefyr* in different clinical specimens is presented in Table 2. Fifty of 69 (72%) patients were hospitalized in two tertiary care hospitals that exclusively cater to immunocompromised/cancer patients. Forty-two patients were females. The largest number of *C*. *kefyr* isolates were obtained from urine samples (n = 31) followed by 24 respiratory samples (sputum, n = 18; tracheal aspirate, n = 4; bronchoalveolar lavage, n = 2). Interestingly, only 5 of 26 (19%) urine isolates but 17 of 24 (71%) respiratory isolates were obtained from male patients (*P* = 0.001) (Table 2). All isolates were negative by germ tube test, formed light purple to lavender-colored colonies on CHROMagar Candida and were identified as *C*. *kefyr* by Vitek2 yeast identification system.

**Table 2:** Distribution of *C*. *kefyr* in different clinical specimens obtained from male and female patients in Kuwait.

Specimen	No. (%) of	Patient characteristics	
type	*C*. *kefyr* isolates	Male	Female
Urine	31 (44.9)	5	26
Sputum	18 (26.1)	15	3
Blood	**4**^b^ **(5.8)**	**2**	**2**
Fluids^a^	**5 (7.2)**	**2**	**3**
Bronchoalveolar lavage	**2 (2.9)**	**1**	**1**
Tracheal aspirate	4 (5.8)	1	3
Vaginal swab	2 (2.9)	0	2
Throat swab	1 (1.5)	0	1
Upper palate swab	1 (1.5)	0	1
Ear swab	1 (1.5)	1	0
Total	69	27	42

^a^Fluids included abdominal drain fluid, n = 2; peritoneal fluid, n = 2 and gastric fluid, n = 1.

^b^*C*. *kefyr* isolates from invasive samples are highlighted in bold.

The molecular identity of all *C*. *kefyr* isolates was confirmed by a simple PCR assay developed in this study. PCR amplification performed with CKEF and CKER primers yielded an amplicon of 268 bp with genomic DNA from *C*. *kefyr* ATCC28838 (S1 Fig, lane 11). No amplicon was obtained from *C*. *albicans* ATCC56881, *C*. *dubliniensis* CBS7987, *C*. *glabrata* ATCC90030, *C*. *parapsilosis* ATCC22019, *C*. *tropicalis* ATCC34139, *C*. *krusei* ATCC6258, *C*. *orthopsilosis* ATCC96139, *C*. *metapsilosis* ATCC96143, *C*. *guilliermondii* CBS6021 and *C*. *famata* CBS796 (S1 Fig, lanes 1–10, respectively) as expected. No amplification was also obtained in PCR assay with DNA from *C*. *lusitaniae* CBS4413, *C*. *nivariensis* CBS9983, *C*. *bracarensis* CBS10154, or from human cells, as expected. The same PCR assay performed with DNA prepared from all 69 clinical *C*. *kefyr* isolates described in this study also yielded an amplicon of 268 bp which confirmed their identification as *C*. *kefyr*. The identification of all four bloodstream isolates was also confirmed by MALDI-TOF MS.

PCR-sequencing of ITS region of rDNA from 27 selected (including all bloodstream and drug-resistant) isolates also identified all isolates as *C*. *kefyr* as they exhibited >99% sequence identity (0, 1 or 2 nucleotide differences) with corresponding sequence from reference *C*. *kefyr* strains CBS4857, CBS5670, CBS1555 and CBS5669 with GenBank accession nos. CP105058, KY103814, KY103791 and KY103739, respectively. The ITS sequence data also identified only two haplotypes among 27 *C*. *kefyr* isolates. Two bloodstream isolates belonged to haplotype 1 while the remaining 25 isolates (including the remaining two bloodstream isolates) belonged to haplotype 2.

### Antifungal susceptibility and molecular basis of resistance to fluconazole and caspofungin

The data on antifungal susceptibility (MIC distribution, MIC range, MIC_50_, MIC_90_, and No. of resistant isolates) for 63 available *C*. *kefyr* isolates (6 isolates were lost during storage) against four (amphotericin B, fluconazole, voriconazole and caspofungin) antifungal drugs are presented in Table 3. Susceptibility testing for micafungin was only performed for *C*. *kefyr* isolate showing reduced susceptibility to caspofungin to confirm resistance to echinocandins. The bloodstream isolates and 6 of 7 other isolates obtained from invasive samples were susceptible to all antifungal agents tested. Five isolates showed reduced susceptibility to amphotericin B (MIC >1 μg/ml) including one isolate from bronchoalveolar lavage and four of these five isolates were obtained during 2015–2018 (Table 1). One isolate (Kw2153/18) was multidrug-resistant as it also exhibited reduced susceptibility to fluconazole (MIC >256 μg/ml), voriconazole (MIC = 32 μg/ml) and caspofungin (MIC = 0.5 μg/ml). This isolate (Kw2153/18) was also tested against micafungin by Etest and was scored as resistant (MIC = 1 μg/ml). PCR-sequencing of *ERG11* from fluconazole-resistant (Kw2153/18) and one fluconazole-susceptible (Kw3415/15) isolate showed that both isolates contained wild-type sequence of Erg11 protein even though three synonymous mutations were identified in both sequences compared to the reference sequence from *C*. *kefyr* ATCC26548 (GenBank accession no. KF964546). Similarly, few synonymous mutations were also identified, however, amino acid sequences of hotspot-1 and hotspot-2 regions of *FKS1* were same in echinocandin-resistant (Kw2153/18) and eight echinocandin-susceptible isolates. Repeat isolates yielded the same susceptibility pattern as the first isolate from all 11 patients.

**Table 3:** *In vitro* susceptibilities of *C*. *kefyr* isolates to four antifungal agents as determined by Etest.

Antifungal drug	No. of isolates	No. of isolates with minimum inhibitory concentration (MIC^a^) (μg/ml) of											MIC range	MIC_50_	MIC_90_	Tentative breakpoints^b^	No. (%) of resistant isolates
		<0.125	0.19	0.25	0.38	0.5	0.75	1	3	4	32	>64					
Amphotericin B	63	11	7	6	18	8	6	2	0	**1**	**4**	0	0.002–32	0.38	1	>1	5 (7.8)
Fluconazole	63	8	17	8	6	14	4	4	1	0	0	**1**	0.003–256	0.25	0.75	>8	1 (1.6)
Voriconazole	63	62	0	0	0	0	0	0	0	0	**1**	0	0.002–32	0.012	0.032	>1	1 (1.6)
Caspofungin	63	45	17	0	0	**1**^c^	0	0	0	0	0	0	0.008–0.38	0.094	0.19	>0.5	1^c^ (1.6)

^a^MIC, minimum inhibitory concentration.

^b^MIC values defining resistance to antifungal drug.

^c^One isolate was categorized as intermediate for caspofungin, however, it was resistant to micafungin (MIC = 1 μg/ml).

### Genotypic heterogeneity among *C*. *kefyr* isolates

**Fig 1:**
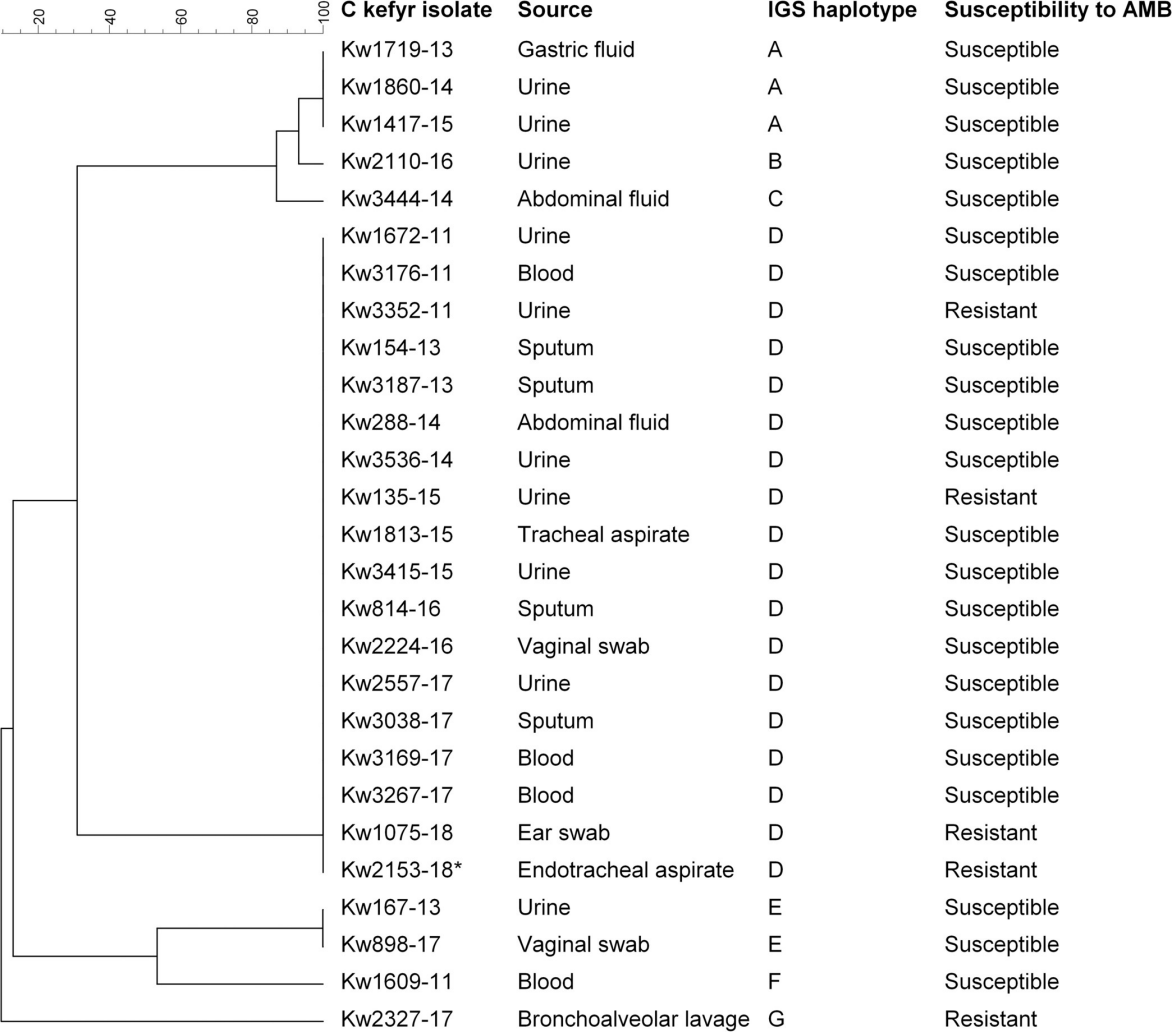
Dendrogram based on unweighted pair group method with arithmetic mean (UPGMA) and derived from intergenic spacer (IGS)-1 sequence data for 27 *C*. *kefyr* isolates. The source of isolation and susceptibility to amphotericin B (AMB) are also shown for each isolate.

Fingerprinting studies with minisatellite-based (M13-MIN) and microsatellite-based (GACA_4_) primers identified only 3 genotypes among all *C*. *kefyr* isolates (data from 7 selected isolates are shown in S2 Fig). PCR-sequencing of IGS1 region from 27 selected isolates identified 7 haplotypes with Haplotype D shared among 18 isolates (Fig 1). However, all Haplotype D isolates were not identical as comparison of sequence data for hotspot-1 and hotspot-2 regions of *FKS1* that was available for nine isolates showed five different patterns.

The DNA sequence data reported in this study have been submitted to EMBL/GenBank database under accession no. LR738859 to LR738911, LR761624 to LR761633 and LR877022 to LR877031.

## Discussion

*C*. *kefyr* is considered as a potential multidrug-resistant yeast species since many isolates exhibit reduced susceptibility to amphotericin B and it also readily develops resistance as a result of short exposure to echinocandins [13, 24, 26–31]. During screening of 8257 yeast isolates collected during an eight-year-period (2011–2018), 69 individual (including 4 candidemia) patient isolates were identified as *C*. *kefyr* with an overall prevalence of 0.83%. During the same period, 1238 culture-confirmed candidemia cases were recorded in Kuwait resulting in a prevalence rate of 0.32% for *C*. *kefyr* fungemia. *C*. *kefyr* is a well-recognized pathogen causing invasive candidiasis among cancer patients and transplant recipients [19, 22–25, 45]. In one study involving patients with hematological malignancies, *C*. *kefyr* was responsible for causing candidemia in nearly 10% patients [24].

Only few studies have specifically investigated the epidemiology of *C*. *kefyr* and its role in invasive disease in hospitalized patients. In a comprehensive recent study based on 20 years (1997–2016) of SENTRY Antifungal Surveillance Program, *C*. *kefyr* was recorded as the 7^th^ most common cause of invasive candidiasis and its prevalence was nearly two times higher (94 of 15308, 0.61%) than in our study [14]. However, this study included patients with candidemia and other invasive *Candida* infections. Interestingly, seven *C*. *kefyr* isolates were also obtained from other invasive samples in Kuwait including two isolates from bronchoalveolar lavage from two patients with pneumonia, two isolates from peritoneal fluid obtained from two patients with peritonitis, two isolates from abdominal fluid from two patients with intra-abdominal infection and one isolate from a gastric aspirate. Although clinical details of patients yielding these isolates were not available, it is pertinent to mention here that 50 of 69 (72%) patients (including 23 cancer patients) yielding *C*. *kefyr* in our study were hospitalized in two tertiary care hospitals where immunocompromised/immunosuppressed patients are treated and isolation of yeast from an invasive sample may be the only sign of a deep-seated fungal infection [6, 46]. Furthermore, *C*. *kefyr* has previously been shown to cause pneumonia, peritonitis, intra-abdominal candidiasis and gastroenteritis in immunocompromised patients [19, 45, 47]. Thus isolation of *C*. *kefyr* from invasive samples from seven patients appears to be clinically significant.

The largest number (31 of 69, 45%) of *C*. *kefyr* isolates were obtained from urine samples mostly (26 of 31, 84%) from female patients and respiratory samples (24 of 69, 34.8%) mostly (17 of 24, 70.8%) obtained from male patients. Candiduria in female patients either results from contamination or reflects a deep-seated infection, particularly in immunocompromised subjects in the intensive care units, which may require invasive procedures for management [48, 49]. Interestingly, repeat urinary isolates were obtained from seven (including six female) patients with one patient yielding five and another patient yielding 10 isolates during several weeks of hospitalization. *C*. *kefyr* was responsible for 2 of 17 (11.8%) cases of urinary tract candidiasis in a recent study from Iran [19] and is also known to cause bladder fungus ball [24]. Similarly, isolation of *Candida* from respiratory samples, particularly sputum or tracheal aspirate may indicate a mere colonization or an invasive infection. *C*. *kefyr* was also recently reported as a cause of nosocomial pneumonia in 5 of 17 (29.4%) hematological patients [45]. It has been postulated that reduced susceptibility of *C*. *kefyr* to antifungal agents that results in its selection during therapeutic and prophylactic use of antifungal drugs (mainly echinocandins) and mucositis caused by anticancer therapy could possibly contribute to increased gastrointestinal colonization and invasion [24, 50–53].

Although the occurrence of *C*. *kefyr* from bloodstream and other invasive samples was nearly same during the two 4-year-periods, four of five isolates with reduced susceptibility to amphotericin B were obtained during last four years. Our data are in agreement with few other studies showing reduced susceptibility of *C*. *kefyr* to amphotericin B [24, 26–29, 54] but contrary to the data reported from Spain [55]. Interestingly, one amphotericin B-resistant isolate was potentially a multidrug-resistant *C*. *kefyr* as it also exhibited reduced susceptibility to both triazoles (fluconazole and voriconazole) and the two echinocandins (caspofungin and micafungin) tested. Four of five bloodstream *C*. *kefyr* isolates in one study involving leukemia patients were resistant to both amphotericin B and caspofungin and two isolates were additionally resistant to fluconazole [29]. Another multidrug-resistant *C*. *kefyr* isolate from a patient with hematologic malignancy has also been described that was not only resistant to fluconazole, amphotericin B and micafungin but was also resistant to flucytosine [24]. Furthermore, five episodes of breakthrough infection occurred; three among patients receiving micafungin and two among patients receiving amphotericin B [24]. Thus *C*. *kefyr* is another emerging potentially multidrug-resistant yeast pathogen in Kuwait, in addition to *C*. *auris*, in recent years [32]. This is a matter of concern since mortality rates for *C*. *kefyr* invasive infections caused by even drug-susceptible strains are higher than those for *C*. *albicans* [50, 52, 53]. This is also evident from the fact that both patients with *C*. *kefyr* candidemia in Kuwait for whom clinical details and outcome were available (clinical details and outcome were not available for the other two candidemia patients) died.

PCR sequencing of *ERG11* revealed wild-type sequence for Erg11 protein in our triazole-resistant *C*. *kefyr* isolate (Kw2153/18). On the contrary, Couzigou et al. [39], reported two non-synonymous mutations (E123Q and K151E) conferring resistance to both fluconazole and voriconazole in a triazole-resistant *C*. *kefyr* isolate in France. Thus the molecular basis of resistance to triazoles in our isolate could either involve upregulation of *ERG11* or overexpression of ABC efflux transporters [12, 41]. PCR-sequencing also did not identify any non-synonymous mutation in hotspot-1 or hotspot-2 region of *FKS1* gene in echinocandin-resistant isolate (Kw2153/18). Again our results are contrary to other studies which have reported mutations in hotspot-1 [31] or hotspot-2 [14] of *FKS1* in echinocandin-resistant *C*. *kefyr* isolates. Taken together, our findings suggest that the multidrug-resistant isolate (Kw2153/18) likely resulted from mechanisms that confer resistance to multiple drugs [12, 41, 56].

Fingerprinting of *C*. *kefyr* isolates was performed to determine clonality. Studies with minisatellite-based and microsatellite-based primers identified only 3 genotypes. PCR sequencing of ITS region of rDNA also identified only two haplotypes among 27 selected isolates. This is similar to highly clonal *Candida* species such as *Candida auris*, *C*. *haemulonii*, *C*. *parapsilosis* and *C*. *orthopsilosis* [15, 32, 34] but contrary to many ITS haplotypes that were identified among isolates of *C*. *dubliniensis*, *C*. *lusitaniae* or *C*. *glabrata* in previous studies [57–59]. Since no multilocus sequence typing scheme is currently available for *C*. *kefyr*, further fingerprinting was performed by PCR-sequencing of IGS1 region of rDNA and identified 7 haplotypes among 27 selected isolates. However, all isolates (n = 18) in the largest cluster (IGS Haplotype D) were also not genotypically identical as further analysis of nine isolates using ITS region of rDNA and hotspot-1 and hotspot-2 regions of *FKS1* gene showed five distinct genotypes. Taken together, our data show that invasive and amphotericin B-resistant *C*. *kefyr* isolates in Kuwait were genetically different strains.

Our study has few limitations. i) The antifungal susceptibility data is based on Etest instead of the reference broth microdilution method and susceptibility testing for micafungin was only performed for *C*. *kefyr* isolate showing reduced susceptibility to caspofungin. ii) Molecular fingerprinting of *C*. *kefyr* isolates by PCR-sequencing of IGS1 region of rDNA was performed for only 27 selected isolates.

## Conclusions

Molecular characterization, antifungal susceptibility profile and genotypic heterogeneity was determined among a large collection of *C*. *kefyr* strains isolated from clinical specimens including four bloodstream and seven other invasive samples collected during 2011–2018 in Kuwait. Four isolates showed reduced susceptibility to amphotericin B and one isolate to all (amphotericin B, fluconazole, voriconazole and caspofungin/micafungin) antifungals tested. Four of five isolates with reduced susceptibility to amphotericin B (including multidrug-resistant isolate) were obtained during last four years suggesting that drug resistance to common antifungals in *C*. *kefyr* is increasing. Furthermore, the invasive and amphotericin B-resistant isolates were genotypically heterogeneous ruling out the possibility of spreading of a dominant and invasive *C*. *kefyr* strain in Kuwait.

## Supporting information

S1 Fig Agarose gel of PCR amplified products using *C*. *kefyr*-specific CKEF and CKER primers and template DNA from reference strain of *C*. *albicans*, *C*. *dubliniensis*, *C*. *glabrata*, *C*. *parapsilosis*, *C*. *tropicalis*, *C*. *krusei*, *C*. *orthopsilosis*, *C*. *metapsilosis*, *C*. *guilliermondii*, *C*. *famata* and *C*. *kefyr* (lanes 1–11, respectively). (DOCX)

S2 Fig Agarose gel of PCR amplicons obtained with GACA-MIC primer (panel A) and M13-MIN primer (panel B). In panel A and B, DNA samples were used from isolate Kw1417/15 (lane 1), Kw3176/11 (lane 2), Kw2327/17 (lane 3), Kw2327/17 (lane 4) (repeat sample), Kw3267/17 (lane 5), Kw2153/18 (lane 6), Kw3352/11 (lane 7), and Kw3169/17 (lane 8).
(DOCX)

S1 Raw images (PDF)
