# Supplementary material for: Candida kefyr in Kuwait: Prevalence, antifungal drug susceptibility and genotypic heterogeneity
Source: PLoS One. 2020 Oct 27;15(10):e0240426. doi: 10.1371/journal.pone.0240426 (PMC7591085; doi:10.1371/journal.pone.0240426)
Supplement: S1 Fig — (DOCX) [file pone.0240426.s001.docx]

**S1 Fig. Agarose gel of PCR amplified products using *C. kefyr*-specific CKEF and CKER primers and template DNA from reference strain of *C. albicans*, *C. dubliniensis*, *C. glabrata*, *C. parapsilosis*, *C. tropicalis*, *C. krusei*, *C. orthopsilosis*, *C. metapsilosis*, *C. guilliermondii,* *C.* *famata* and *C. kefyr* (lanes 1-11, respectively).** Lane M is 100 bp DNA marker and the position of migration of 100 bp, 300 bp and 600 bp fragments are marked.
