# Supplementary material for: Candida kefyr in Kuwait: Prevalence, antifungal drug susceptibility and genotypic heterogeneity
Source: PLoS One. 2020 Oct 27;15(10):e0240426. doi: 10.1371/journal.pone.0240426 (PMC7591085; doi:10.1371/journal.pone.0240426)
Supplement: S2 Fig — Agarose gel of PCR amplicons obtained with GACA-MIC primer (panel A) and M13-MIN primer (panel B). In panel A and B, DNA samples were used from isolate Kw1417/15 (lane 1), Kw3176/11 (lane 2), Kw2327/17 (lane 3), Kw2327/17 (lane 4) (repeat sample), Kw3267/17 (lane 5), Kw2153/18 (lane 6), Kw3352/11 (lane 7), and Kw3169/17 (lane 8). (DOCX) [file pone.0240426.s002.docx]

**S2 Fig. Agarose gel of PCR amplicons obtained with GACA-MIC primer (panel A) and M13-MIN primer (panel B). In panel A and B, DNA samples were used from isolate Kw1417/15 (lane 1), Kw3176/11 (lane 2), Kw2327/17 (lane 3), Kw2327/17 (lane 4) (repeat sample), Kw3267/17 (lane 5), Kw2153/18 (lane 6), Kw3352/11 (lane 7), and Kw3169/17 (lane 8).** Lane M is 100 bp DNA ladder and the positions of migration of 300 bp, 600 bp and 1500 bp fragments are marked.
