# Supplementary material for: Candida kefyr in Kuwait: Prevalence, antifungal drug susceptibility and genotypic heterogeneity
Source: PLoS One. 2020 Oct 27;15(10):e0240426. doi: 10.1371/journal.pone.0240426 (PMC7591085; doi:10.1371/journal.pone.0240426)

Original raw figures for Fig. S1, Fig. S2, Panel A and Fig. S2, Panel B

Original of Fig. S1

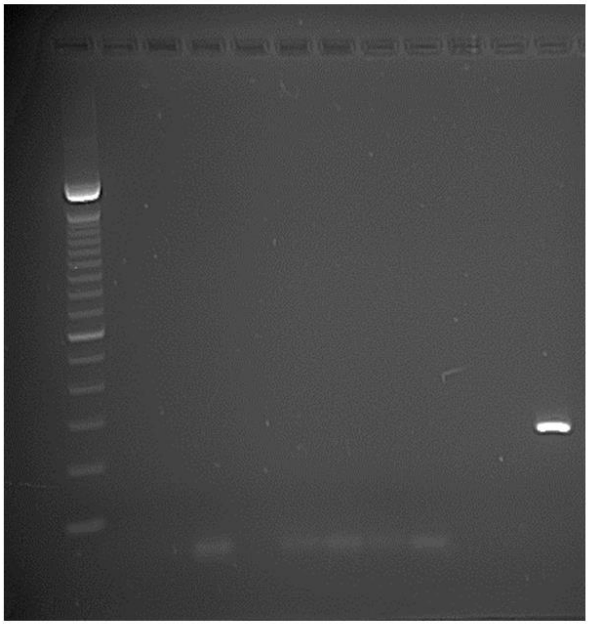

Original of Fig. S2, Panel A

GACA-MIC

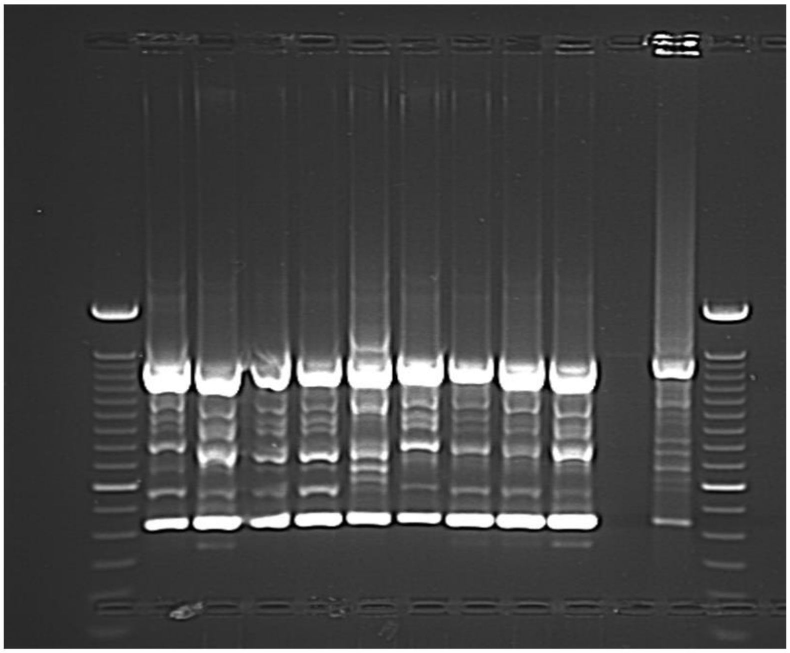

Original of Fig. S2, Panel B

M13-MIN

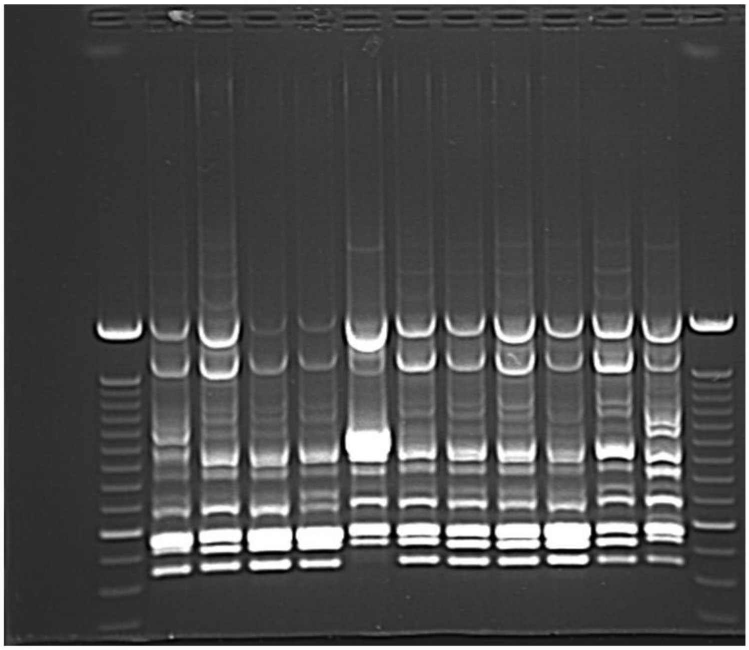

Supplement: S1 Raw images — (PDF) [file pone.0240426.s003.pdf]
